# Cost-effectiveness of recurrence risk guided care versus care as usual in women who suffered from early-onset preeclampsia including HELLP syndrome in their previous pregnancy (the PreCare study)

**DOI:** 10.1186/1471-2393-10-60

**Published:** 2010-10-11

**Authors:** Denise HJ Delahaije, Sander MJ van Kuijk, Carmen D Dirksen, Simone JS Sep, Louis L Peeters, Marc E Spaanderman, Hein W Bruinse, Laura D de Wit-Zuurendonk, Joris AM van der Post, Johannes J Duvekot, Jim van Eyck, Mariëlle G van Pampus, Mark ABHM van der Hoeven, Luc J Smits

**Affiliations:** 1 Department of Clinical Epidemiology and Medical Technology Assessment, Maastricht University Medical Centre, Maastricht, The Netherlands; 2 Department of Obstetrics and Gynaecology, Maastricht University Medical Centre, Maastricht, The Netherlands; 3 Department of Epidemiology, Maastricht University, Maastricht, The Netherlands; 4 Department of Obstetrics and Gynaecology, University Medical Centre St. Radboud, Nijmegen, The Netherlands; 5 Department of Obstetrics and Gynaecology, University Medical Centre Utrecht, The Netherlands; 6 Department of Obstetrics and Gynaecology, Máxima Medical Centre, Veldhoven, The Netherlands; 7 Department of Obstetrics and Gynaecology, Academic Medical Centre, Amsterdam, The Netherlands; 8 Department of Obstetrics and Gynaecology, Erasmus Medical Centre, Rotterdam, The Netherlands; 9 Department of Obstetrics and Gynaecology, Isala Clinics, Zwolle, The Netherlands; 10 Department of Obstetrics and Gynaecology, University Medical Centre, Groningen, The Netherlands; 11 Department of Pediatrics, Maastricht University Medical Centre, Maastricht, The Netherlands

## Abstract

**Background:**

Preeclampsia and HELLP syndrome may have serious consequences for both mother and fetus. Women who have suffered from preeclampsia or the HELLP syndrome, have an increased risk of developing preeclampsia in a subsequent pregnancy. However, most women will develop no or only minor complications. In this study, we intend to determine cost-effectiveness of recurrence risk guided care versus care as usual in pregnant women with a history of early-onset preeclampsia.

**Methods/design:**

We developed a prediction model to estimate the individual risk of recurrence of early-onset preeclampsia and the HELLP syndrome. In a before-after study, pregnant women with preeclampsia or HELLP syndrome in their previous pregnancy receiving care as usual (before introduction of the prediction model) will be compared with women receiving recurrence risk guided care (after introduction of the prediction model).

Eligible and pregnant women will be recruited at six university hospitals and seven large non-university tertiary referral hospitals in the Netherlands.

The primary outcome measure is the recurrence of early-onset preeclampsia or HELLP syndrome in women allocated to the regular monitoring group.

For the economic evaluation, a modelling approach will be used. Costs and effects of recurrence risk guided care with those of care as usual will be compared by means of a decision model. Two incremental cost-effectiveness ratios will be calculated: 1) cost per Quality Adjusted Life Year (mother unit of analysis) and 2) cost per live born child (child unit of analysis).

**Discussion:**

This is, to our knowledge, the first study that evaluates prospectively the efficacy of a multivariable prediction rule for recurrent hypertensive disease in pregnancy. Results of this study could either be integrated into the current guideline on Hypertensive Disorders in Pregnancy, or be used to develop a new guideline.

## Background

### The disease

Preeclampsia is defined as de novo development of hypertension (> 140/90 mmHg) in combination with proteinuria after 20 weeks of gestation in pregnant women [1]. One of the most severe forms of this disease is called the HELLP syndrome, which is derived from the acronyms of the symptoms (Hemolysis, Elevated Liver enzymes and Low Platelets).

Preeclampsia (also termed early-onset preeclampsia) and HELLP syndrome may be life-threatening for both mother and child [2]. In the mother, these disorders predispose to premature cardiovascular disorders such as chronic hypertension, ischemic heart disease and stroke later in life [3]. Studies in children, born after preeclamptic pregnancies and who were relatively small at birth, have shown an increased risk of stroke in adult life, an increased risk of coronary heart disease and metabolic syndrome [4-6]. Preeclampsia and especially HELLP syndrome are perceived by patients and their partners as a highly traumatic life event, both during and after pregnancy [7].

In the United States and Europe, the prevalence of clinically relevant preeclampsia is approximately 2% of all pregnancies, with HELLP syndrome complicating preeclampsia approximately in 10 to 20% of the cases [3,8]. Maternal mortality rate in the Netherlands due to hypertensive disease during pregnancy amounts to 4.0 per 100,000 live births in 1993-2002 [9].

Although several risk factors have been identified, it is difficult to develop effective strategies for the prevention and treatment of these disorders [3]. Strategies applied nowadays are diverse and include antenatal surveillance, modification of lifestyle, dietary interventions and pharmacological therapy. In the last two decades, clinical guidelines for the management of preeclampsia and HELLP syndrome have been adopted in most developed countries [10-12].

Clinical management of preeclampsia or HELLP syndrome depends on the gestational age at onset, severity of symptoms, laboratory abnormalities, size and condition of the unborn infant, disease progression and response to symptomatic treatment.

Mild preeclampsia is usually managed expectantly, as opposed to the management of severe preeclampsia which is more pro-active consisting of the administration or magnesium sulphate, often combined with antihypertensives [13]. When gestational age is less than 34 weeks and both maternal and fetal condition are satisfactory, it is usually recommended to prolong pregnancy for at least 48 hours to benefit optimally from the enhancing effect of corticosteroid administration on fetal lung maturation. Fetal surveillance is an important component of the management. Delivery is the only causal treatment and therefore, the management of choice from 37 weeks' gestation onward [14,15].

### The health care problem

Although preeclampsia and HELLP syndrome are considered diseases of the first pregnancy, the risk of developing recurrent preeclampsia or HELLP syndrome is increased among parous women with preeclampsia and/or HELLP syndrome in their previous pregnancy. Sep et al. [16] conducted a literature search in order to identify prediction tests for recurrent disease. The recurrence rates reported vary from none to 31 percent for preeclampsia and from 3 percent to 7 percent for HELLP syndrome. Fortunately, the majority of women with a history of preeclampsia or HELLP syndrome have uncomplicated subsequent pregnancies.

To the best of our knowledge there is at this moment no consensus about the management of pregnant women with a history of early-onset preeclampsia or HELLP syndrome.

As a result, follow-up and counselling of these patients varies per centre, gynaecologist and patient, and is largely based on the perceived risk by the responsible care provider of recurrence of the disease in the next pregnancy and the level of anxiety of the patient and its resulting demand for care. Formerly preeclamptic patients are often subjected to various medical screening programmes to detect associated disorders, followed by additional exams by other specialists or the initiation of some management (postpartum evaluation). During a next pregnancy, care varies from regular surveillance by the gynaecologist to intensive surveillance and counselling in order to identify the onset of adverse pregnancy course as early as possible.

### Motivation and relevance for the study

This lack of uniformity in the treatment of these patients asks for more standardisation, e.g. by providing care tailored to the individualised risk assessment.

Current policy may not be efficient. Since only a small percentage of these women develop early-onset recurrent disease in their next pregnancy, clinical management in the next pregnancy may benefit from subdividing these women into subgroups with or without increased risk. The care provided to these women can then be adjusted to their actual risk profile. Particularly former patients at low risk are expected to benefit from this type of risk stratification. Recurrence risk guided care could lead to a substantial reduction of (health care) costs and an increased quality of care.

We have recently developed a multiple-factor model for the prediction of recurrent early-onset preeclampsia and/or HELLP syndrome during the current pregnancy in women with a prior pregnancy complicated by preeclampsia and/or HELLP syndrome (van Kuijk SMJ, Nijdam ME, Janssen KJ, Sep SJ, Peeters LL, Delahaije DHJ, Spaanderman ME, Bruinse HW, Franx A, van Rijn BB, Bots ML, Langenveld J, van der Post AM, Smits LJ: A preconceptional prediction model for recurrent early-onset preeclampsia and the HELLP syndrome, unpublished).

The PreCare study (pregnant women with previous preeclampsia: efficiency of care based on recurrence risk estimation), was designed to (1) estimate cost-effectiveness of recurrence risk guided care versus care as usual for pregnant women with a previous pregnancy being complicated by preeclampsia or HELLP syndrome and (2) to validate the prediction model externally on the basis of the results of a prospective cohort and to update the prediction model if necessary. To this end, we will use the prediction model to differentiate the intensity of monitoring of pregnant women with a history of early-onset preeclampsia and/or HELLP syndrome. Patients are assigned to either the 'regular monitoring' or 'intensive monitoring' protocol. We will compare this strategy, referred to as recurrence risk guided care, with usual care for these patients in The Netherlands.

For this purpose, the following research questions were specified:

1. What are the effects of recurrence risk guided care versus care as usual for pregnant women with a previous preeclamptic pregnancy, on maternal and fetal morbidity and mortality?

2. What are the effects of recurrence risk guided care versus care as usual on specific and generic quality of life, anxiety, depression and development of posttraumatic stress?

3. What are the societal costs associated with the effects of recurrence risk guided care versus care as usual?

4. What is the cost-effectiveness of recurrence risk guided care versus care as usual?

### Development of prediction model

Although earlier studies have identified individual predictive factors for recurrent preeclampsia, a combination of variables for the prediction of recurrent preeclampsia has not been explored until recently. A simple prepregnant prediction rule which includes several predictive factors was developed by Sep et al. [17]. Unfortunately, patient data were collected from a single hospital and the number of included patients was limited (n = 186).

We developed a prediction model to estimate the individual recurrence risk of recurrence of early-onset preeclampsia and/or the HELLP syndrome. We used data of 407 women with early-onset preeclampsia and/or the HELLP syndrome in their first pregnancy, who had undergone subsequent postpartum screening and who had a recorded consecutive ongoing pregnancy for which maternal and neonatal outcomes were available. Data were collected from four university hospitals (Maastricht University Medical Centre, University Medical Centre Utrecht, Academic Medical Centre Amsterdam and University Medical Centre St. Radboud Nijmegen), and one tertiary referral hospital (Máxima Medical Centre Veldhoven).

Predictors of recurrent disease were preselected based on availability in the five different hospitals, previous literature and gynaecologists' expert opinion. Fasting circulating level of glucose measured at postpartum screening, gestational age at delivery of the first pregnancy, prior small-for-gestational-age newborn, chronic hypertension and maternal BMI before the second pregnancy proved to be the predictors within a logistic regression model (van Kuijk SMJ, Nijdam ME, Janssen KJ, Sep SJ, Peeters LL, Delahaije DHJ, Spaanderman ME, Bruinse HW, Franx A, van Rijn BB, Bots ML, Langenveld J, van der Post AM, Smits LJ: A preconceptional prediction model for recurrent early-onset preeclampsia and the HELLP syndrome, unpublished).

Since a model tends to perform best in the derivation sample, called 'overfitting', we used bootstrapping techniques to internally validate the model. A shrinkage factor was computed to shrink the regression coefficients in order to get a more conservative risk estimate.

## Methods/design

### Design

The PreCare study uses a before-after design, in which outcomes and costs before the introduction of the prediction model (i.e. care as usual) are compared with outcomes and costs after introduction of the prediction model (i.e. recurrence risk guided care).

**Figure 1:**
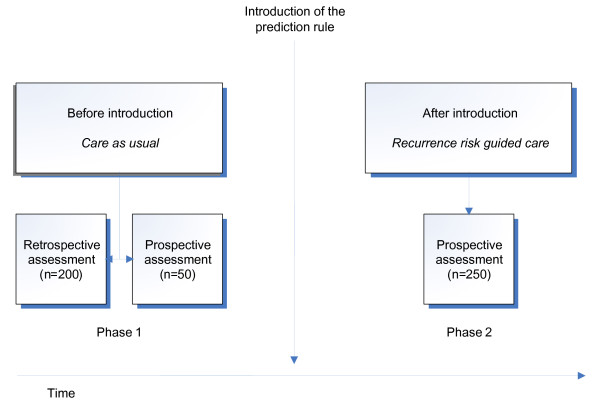
**Design of the study**.

This study consists of two consecutive phases. In the first phase of the study, 50 women receiving care as usual will be followed prospectively from their first pregnancy-related outpatient visit until 3 months post partum. These 50 women will receive questionnaires in order to measure quality of life, anxiety, depression, posttraumatic stress and costs outside the hospital. Retrospective data of an additional group of 200 women who have been treated with care as usual in the past will be collected later on. Then the prediction rule will be introduced. In the second phase, 250 women will receive either protocolised regular monitoring or protocolised intensive monitoring depending on their risk of developing early-onset preeclampsia or HELLP syndrome during their current pregnancy, as estimated on the basis of the prediction model. In regular monitoring, pregnant women are monitored less intensively than in intensive monitoring. Regular monitoring and intensive monitoring are described in detail in the 'Monitoring protocols' section. Figure 1 summarises the design.

### Participants/eligibility criteria

In phase 1 and 2, pregnant women (aged 18 years and older) with preeclampsia and/or HELLP syndrome in their previous pregnancy who have visited the outpatient clinic until 16+6 weeks of pregnancy are eligible for participation in the PreCare study.

Given these general eligibility criteria, two subgroups are distinguished in recurrence risk guided care (phase 2).

In women who gave birth after early-onset preeclampsia or HELLP syndrome until 33+6 weeks gestational age in the previous pregnancy, the prediction model is applied in order to allocate the participants to one of the two monitoring protocols. The data which are required as input parameters for the prediction model should be available. These variables mostly come from the postpartum evaluation.

Women who gave birth between 34 and 36+6 weeks gestational age in the previous pregnancy face a relatively small risk of developing recurrent preeclampsia or HELLP syndrome, compared to women who gave birth before 34 weeks. Therefore, they all are allocated to the regular monitoring subgroup [18].

Women suffering from severe co-morbidity, such as diabetes mellitus, SLE, renal disease, cardiac disease or anti-phospholipids syndrome are excluded from the study as they will all receive (or have received where it concerns the retrospective patients) intensive surveillance. The current study will not include women who gave birth after 37 weeks gestational age.

The study population is recruited from six university hospitals and seven large non-university tertiary referral hospitals in the Netherlands: the Maastricht University Medical Centre, University Medical Centre St. Radboud Nijmegen, the Isala Clinics Zwolle, Erasmus Medical Centre Rotterdam, Academic Medical Centre Amsterdam, University Medical Centre Utrecht, Máxima Medical Centre Veldhoven, the University Medical Centre Groningen, Martini Hospital Groningen, Amphia Hospital Breda, Atrium Medical Centre Heerlen, Jeroen Bosch Hospital Den Bosch and Kennemer Hospital Haarlem.

### Recruitment procedures

For the prospective assessment, 50 care as usual patients and 250 recurrence risk guided patients will be included in the study.

The research nurse or the gynaecologist of participating hospitals identifies eligible women by screening the appointment system of the outpatient clinic. After the pregnant woman has given informed consent for participation in the study, patients in phase 1 will be enrolled in care as usual whereas patients in phase 2 will be enrolled in recurrence risk guided care.

In recurrence risk guided care (phase 2), a simple web based risk calculator for the determination of recurrence risk and required intensity of surveillance has been developed and has been made accessible to gynaecologists and research nurses.

Information about the recommended components of intensive monitoring and regular monitoring has been provided together with the risk calculator.

For the retrospective assessment, 200 patients who were treated according to care as usual will be used as a comparator group based on matching. Matching criteria will at least include maternal age (difference < 5 years) and gestational age at delivery. Consequently, at study completion, there will be 250 care as usual patients who will be compared with 250 recurrence risk guided patients.

### Data collection

For the prospective assessment (n = 50 care as usual; n = 250 recurrence risk guided care), baseline demographics, past obstetric and medical history will be recorded in case report forms. Information is obtained on the condition of mother and child, infant weight, infant length, morbidity and mortality from the infant and maternal records. If applicable, details of the admittance of the child to the neonatal intensive care, high care or medium care unit will also be collected. All activities in the hospital will be documented until three months postpartum.

We will assess not only clinical outcomes, but also health-related quality of life by using the EQ-5 D [19], anxiety by using the STAI [20], depression by using the Beck Depression Inventory [21] and posttraumatic stress by using the PTSD Symptom Scale [22]. The QoL questionnaires will be administered at 3-month intervals between baseline (before 20 weeks of pregnancy), at 29 weeks of pregnancy, ten days and three months postpartum. Costs outside the hospital, such as GP care, midwife and maternity care, productivity losses and out-of-pocket expenses will be obtained by means of a retrospective cost questionnaire with a recall period of 3 months. Long-term follow up of the women and children may be possible, but is not included in the current study.

For women enrolled in the retrospective assessment (n = 200), all data described above will be collected, except data on health-related quality of life, anxiety, depression, posttraumatic stress and costs outside the hospital.

### Monitoring protocols

#### Development of the protocols

All gynaecologists of the participating centres were approached and they participated in a consensus process, during which the final protocols were designed. Nearly all were obstetricians specialised in hypertensive disorders of pregnancy. After reaching consensus, the protocols were distributed among all providers of care in the study.

#### The protocols

**Figure 2:**
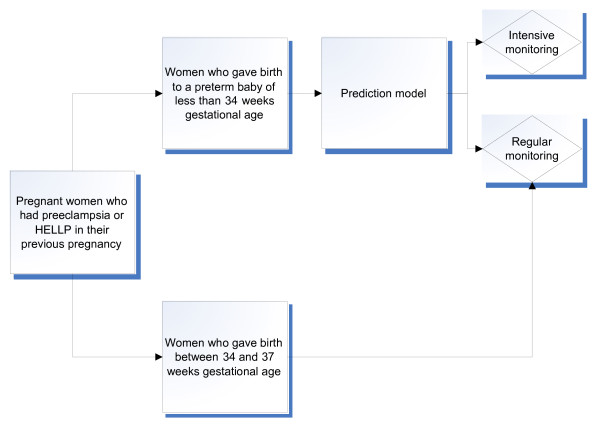
**Recurrence risk guided care (phase 2)**.

Figure 2 shows the process of recurrence risk guided care.

#### Regular monitoring

Women will visit the outpatient clinic 9 - 11 times: (8, 12), 16, 20, 24, 28, 32, 36, 38, 39 and 40 weeks gestational age. The visits at 16 and 20 weeks will be scheduled after the first trimester screening procedure and the routine ultrasound screening for structural malformations, respectively. If feasible, six of these eleven visits (12, 20, 28, 36, 39, 40) ought to be performed by the same obstetrician, whereas the remaining five (reassuring) visits (8, 16, 24, 32, 38) may be performed by a nurse-practitioner or may be performed by the gynaecologist of the referring clinic (as of 32 weeks, in regular and intensive monitoring).

#### Intensive monitoring

Women will visit the outpatient clinic 14 - 16 times: (8, 12), 16, 18, 20, 22, 24, 26, 28, 30, 32, 34, 36, 38, 39 and 40 weeks gestational age.

At two occasions during an intensive monitoring pregnancy (16 and 20 weeks), the patient will undergo a series of diagnostic tests, which consist of

1. Measurements in urine: (micro)albuminuria, creatinine, protein (protein serves to calculate the protein-to-creatinine ratio)

2. Measurements in blood: Haemoglobin, platelet count, mean platelet volume, creatinine, urate, CRP, glucose, fibronectin, Flt-1, endoglin

3. 24 h blood pressure monitoring. This assessment is optional (not obligatory)

4. Assessment of the Doppler flow velocity profile in both uterine arteries (at 20 weeks)

Activities other than, or additional to those described in the regular monitoring and intensive monitoring protocols (such as intercurrent admissions, additional outpatient visits or diagnostic testing, or telephone/e-mail contacts) will be registered.

#### Study outcomes

Recurrence risk guided care will be compared with care as usual with respect to several outcomes.

Primary outcome is the occurrence of either early-onset preeclampsia or HELLP syndrome. Early-onset preeclampsia and early-onset HELLP syndrome are defined according to the criteria of the International Society for the Study of Hypertension in Pregnancy with the criterion of delivery before 34 weeks after the last menstrual period [23]. Secondary clinical outcome measures are: gestational age of onset of preeclampsia, eclampsia, HELLP syndrome, intrauterine growth restriction, caesarean section, admission to the neonatal intensive care unit (NICU), gestational age at delivery and maternal/infant mortality. Other secondary outcome measures are societal costs, quality of life, anxiety, depression, development of posttraumatic stress, satisfaction with treatment, protocol adherence and cost-effectiveness.

### Statistical analysis

#### Sample size

Sample size calculation was based on the expected fraction of women assigned to regular monitoring either on the basis of the prediction rule or gestational age at the time of previous delivery (67%), the expected failure rate within this category (2%), a type I error (one-sided) of 0.05 and a type II error of 0.2. In order to be able to exclude failure rates of 5% or more, 150 patients assigned to regular monitoring are needed. This means that results of about 225 patients in the recurrence risk based care are needed. The estimated number of 250 allows for drop out and incomplete data of 25 patients.

#### Data analysis

The primary analysis concerns the incidence of recurrent early-onset preeclampsia or HELLP syndrome in both groups (recurrence risk guided care versus care as usual). Analyses will be adjusted for potential confounders (including demographic factors). Confidence intervals will be adjusted by means of multilevel analysis (hospital).

We will also analyse whether the new patient data indicate the need for an update of the predictive model. For this purpose, we will first compare the characteristics of the original population used for model development with the new population, the so-called "validation population". For example, the incidence of early-onset preeclampsia may differ between the original and validation population. It is also possible that the latter population has a different case-mix (i.e. differences in distributions of the predictors in the population), because, e.g., more hospitals are included. In addition, different predictor-outcome associations and additional predictors, that are not included in the model but are either more or less frequent in the new population, could play a role. We will also assess model performance in the validation population by comparing sensitivity, specificity, positive and negative predictive values and discrimination and calibration compared to the performance in the derivation population.

If the analysis should indicate lower accuracy than expected, we will analyse to what extent all these factors indeed affect the model's accuracy and update the model to obtain adequate accuracy. Updating of the model will be done by means of re-calibration (step 1) and model revision (step 2) [24-26].

#### Economic evaluation

A cost-effectiveness analysis will be performed from a societal perspective, comparing the costs and effects of recurrence risk guided care versus care as usual. The time horizon of the study is 9 months (3 mo amenorrhea - 3 mo post partum). Discounting is not relevant given the short time horizon.

The cost analysis will be performed according to the Dutch guidelines for cost calculations [27]. All hospital resource use and costs associated with care for pregnant women and their newborns will be calculated from study entry until 3 months post partum and include costs such as outpatient visits and hospital admissions. Cost prices will be obtained from participating hospitals. If prices are not readily available, directive prices will be used [27] or additional calculations will be made. Costs in the analysis also include direct non-health care costs (travel costs) and indirect costs (productivity loss).

Effect parameters are clinical outcomes, health related quality of life, anxiety, depression and posttraumatic stress.

Currently, no economic evaluation methods are available that integrate health outcomes of both mother and child into a single outcome measure. However, preeclampsia may have health effects for both the pregnant women and her (unborn) child. Therefore, the main cost-effectiveness analysis will involve calculating two incremental cost-effectiveness ratios (ICERs) expressing 1) the cost per Quality Adjusted Life Year (QALY, mother unit of analysis) and 2) the cost per live born infant (child unit of analysis). To this end, a decision model will be developed. Modelling in economic evaluation is considered useful for example when experimental observations from a trial are missing, which in this study applies to some parameters in the care as usual group [28,29].

#### Ethical considerations

This study has been approved by the ethical committee of the University Hospital Maastricht (Ref.no. MEC 07-2-078). A total of six academic and seven non-academic hospitals participate in the study; all of them have completed their obligatory feasibility assessment procedure successfully. Informed consent is being obtained from all patients prior to enrolment into the study.

## Discussion

This study is expected to yield information on health outcomes and costs of adjusting the level of care to the estimated probability of recurrent preeclampsia. The results can provide a basis for more uniform (and evidence-based) guidelines for care for formerly preeclamptic women and possibly lead to more cost-effective provision of health care. With respect to health care costs, it is expected that mean costs per patient will decrease as a result of a reduction in intensive maternal and fetal surveillance. Potential savings can be even higher, since the majority of women assigned to regular monitoring may be adequately served by care provided by in-hospital midwifes [30].

The close cooperation with many centres enables us to reach a representative study population of pregnant women who have experienced preeclampsia in their previous pregnancy, which enhances the applicability of the results to all former preeclamptic women. Results of this study will be disseminated by means of presentations at scientific meetings and peer-reviewed publications. Study outcomes will also be communicated directly to the NVOG (Dutch Association of Obstetrics and Gynaecology), KNOV (Royal Dutch Association of Midwives), and the Dutch HELLP syndrome foundation. In addition, we will cooperate with the NVOG in order to produce recommendations for the formulation of guidelines. The recommendations could either be integrated into the current guideline on Hypertensive Disorders in Pregnancy, or be used to develop a new guideline. The results of this study will be used to standardise the postpartum evaluation of women with a recent history of preeclampsia or HELLP syndrome. It is expected that the number of tests in the postpartum evaluation will not only be reduced, but also synchronised.

A randomised controlled trial design is usually preferred over any other design. However, for this study such a design was not considered applicable. A before-after study was chosen (instead of a fully prospective, randomised study) because of the risk of care as usual being contaminated with the regular monitoring and intensive monitoring protocols. Because current care as usual is not standardised, and blinding of the participating gynaecologists with respect to predictive factors for recurrent disease is practically unfeasible, a randomised design could reduce the contrast between study arms and thereby threaten the validity of the results.

As the care provided to women assigned to regular monitoring is less intense, a possible consequence may be that the detection of clinical signs of preeclampsia or HELLP syndrome is somewhat later than in the current care as usual approach. However, we do not expect this to lead to adverse maternal or fetal outcomes, because the prediction model suggests that the recurrence rate in this group is low (< 1%) whereas there is also increased alertness for early signs of pregnancy complications in women with a history of preeclampsia or HELLP syndrome.

In summary, the PreCare study is designed to provide information on whether recurrence risk guided care is a worthwhile strategy compared to current care for pregnant women who suffered from preeclampsia or HELLP syndrome during their previous pregnancy.

## Competing interests

The authors declare that they have no competing interests.

## Authors' contributions

DD drafted the manuscript; LS, CD, LP, SS participated in the design of the study; SK was helpful in writing the section concerning the prediction model. All authors discussed and fine tuned the final study design. They are participating in the acquisition of data. Finally, all authors read and approved the final manuscript.

## Pre-publication history

The pre-publication history for this paper can be accessed here:

http://www.biomedcentral.com/1471-2393/10/60/prepub
